# Differences in gut microbiome by insulin sensitivity status in Black and White women of the National Growth and Health Study (NGHS): A pilot study

**DOI:** 10.1371/journal.pone.0259889

**Published:** 2022-01-19

**Authors:** Candice A. Price, Guillaume Jospin, Kristy Brownell, Jonathan A. Eisen, Barbara Laraia, Elissa S. Epel

**Affiliations:** 1 Department of Molecular Biosciences, School of Veterinary Medicine, University of California, Davis, CA, United States of America; 2 Genome Center, University of California Davis, Davis, CA, United States of America; 3 Center for Obesity Assessment, Study and Treatment, University of California, San Francisco, California, United States of America; 4 School of Public Health, University of California, Berkeley, California, United States of America; 5 Department of Evolution and Ecology, University of California, Davis, CA, United States of America; 6 Department of Medical Microbiology and Immunology, University of California, Davis, CA, United States of America; 7 Department of Psychiatry, University of California, San Francisco, CA, United States of America; University of Illinois Urbana-Champaign, UNITED STATES

## Abstract

The prevalence of overweight and obesity is greatest amongst Black women in the U.S., contributing to disproportionately higher type 2 diabetes prevalence compared to White women. Insulin resistance, independent of body mass index, tends to be greater in Black compared to White women, yet the mechanisms to explain these differences are not completely understood. The gut microbiome is implicated in the pathophysiology of obesity, insulin resistance and cardiometabolic disease. Only two studies have examined race differences in Black and White women, however none characterizing the gut microbiome based on insulin sensitivity by race and sex. Our objective was to determine if gut microbiome profiles differ between Black and White women and if so, determine if these race differences persisted when accounting for insulin sensitivity status. In a pilot cross-sectional analysis, we measured the relative abundance of bacteria in fecal samples collected from a subset of 168 Black (*n* = 94) and White (*n* = 74) women of the National Growth and Health Study (NGHS). We conducted analyses by self-identified race and by race plus insulin sensitivity status (e.g. insulin sensitive versus insulin resistant as determined by HOMA-IR). A greater proportion of Black women were classified as IR (50%) compared to White women (30%). Alpha diversity did not differ by race nor by race and insulin sensitivity status. Beta diversity at the family level was significantly different by race (p = 0.033) and by the combination of race plus insulin sensitivity (p = 0.038). Black women, regardless of insulin sensitivity, had a greater relative abundance of the phylum *Actinobacteria* (p = 0.003), compared to White women. There was an interaction between race and insulin sensitivity for *Verrucomicrobia* (p = 0.008), where among those with insulin resistance, Black women had four fold higher abundance than White women. At the family level, we observed significant interactions between race and insulin sensitivity for *Lachnospiraceae* (p = 0.007) and *Clostridiales Family XIII* (p = 0.01). Our findings suggest that the gut microbiome, particularly lower beta diversity and greater *Actinobacteria*, one of the most abundant species, may play an important role in driving cardiometabolic health disparities of Black women, indicating an influence of social and environmental factors on the gut microbiome.

## Introduction

Type 2 diabetes (T2D) prevalence in Blacks is almost twice that of Whites [1]. It is expected that almost half of Black women in the U.S. will develop diabetes [2]. This risk is greater in Black women than Black men and other race/ethnicities [3], in part due to Black women having a high prevalence of overweight and obesity [4]. Obesity promotes increased inflammation and insulin resistance. Greater insulin resistance is reported in Black women compared to White women in some [5, 6], but not all studies [7–9], even when matched for BMI. However, the mechanisms contributing to the progression of insulin resistance in Black women are not well-understood [7, 10, 11]. Ancestral genetic differences have been linked to insulin resistance in Black individuals [12, 13], but the recognition of race as a social construct highlights social determinants of health as important mediators [14].

Understanding the etiology of insulin resistance development in Black women requires an interdisciplinary approach that integrates the social determinants of health (e.g. environmental factors) with physiological outcomes. One such approach is the understanding the role of the gut microbiome in the pathway of cardiometabolic disease development. The gut microbiome is influenced largely by environmental and social factors, such as diet and psychological stress [15–17], and is linked to the development of insulin resistance and T2D [18–20].

The three most predominant gut bacteria species at the phylum level are *Firmicutes*, *Bacteroidetes*, *and Actinobacteria* [21–24]. Greater *Firmicutes* and *Actinobacteria* but lower *Bacteroidetes* characterize microbial communities in obesity and T2D [25–27]; although this finding is not consistent amongst studies [28]. At the genus level *Ruminococcus*, *Blautia* (of the *Firmicutes* phylum) and *Fusobactria* (of the *Fusiobacteria* phylum) and are positively associated with T2D, whereas *Bifidobacteria* and *Bacteroides* (of the *Actinobacteria* and *Bacteroidetes* phylum, respectively) appear to be protective against T2D [19]. Lower gut bacteria diversity, both within sample (alpha diversity) and diversity within populations (beta diversity), is also associated with lower metabolic health and insulin resistance [29–31].

Bacteria diversity differs amongst ethnic groups [32]. Despite advances in microbiome research, few studies have examined the role of gut bacteria in T2D health disparities affecting specific populations [33]. Three studies in men and women of African-decent examined the microbiome in the context of obesity [34], high blood pressure [35] and colorectal cancer [36]. Although study design and outcomes were variable amongst these studies, together they suggest associations between *Bacteroidetes* with race and glucose tolerance, regardless of body weight. One study observed a greater ratio of *Firmicutes* to *Bacteroidetes* in Blacks versus other race and ethnicities [36]. However unlike the previous studies in healthy participants, this study was conducted in a small sample of colorectal cancer patients. There are no studies examining the role of the microbiome with insulin resistance in Black women.

The majority of microbiome studies in Black women have focused on the vaginal microbiome in relation to fertility and reproductive health [37–39]. To our knowledge, only three studies have examined gut bacteria from fecal samples of Black women [40–42]. Of these, only one compared bacteria profiles between overweight, pre- and post-menopausal, Black and White women. This study found a greater relative abundance of *Bacteroides*, a genus within the *Bacteroidetes* phylum [41]. Comparisons between Black women in the U.S. versus lean Ghanaian women found *Bacteroides* and family *Lachnospiraceae* to be higher in U.S. Black women [40]. This was also accompanied by differences in beta diversity between groups and lower alpha diversity in U.S. Black women. Carson and colleagues observed racial differences in bacteria beta diversity (between race groups) but no differences for within sample alpha diversity [41]. Similar findings were observed in postmenopausal Black and White women [42]. Only one study has linked beta diversity, *Bacteroidetes* or *Lachnospiraceae* to obesity and insulin resistance in Black women, however this study compared ethnic differences (U.S.-living vs Ghana-living). No studies in the U.S. have investigated these relationhips by sex and race differences to help explain racial disparities in health.

The gut microbiome may provide us greater insight into furthering our understanding of obesity and type 2 diabetes risk in Black women. Given the paucity of literature on this topic, we collected fecal samples from Black and White women of the National Growth and Health Study (NGHS) to explore whether or not gut bacteria profiles differed by race and insulin sensitivity status. We hypothesized that we would identify gut bacteria profiles, specifically lower *Firmicutes* to *Bacteroidetes* ratio and lower alpha diversity that would be associated with greater insulin resistance in Black women.

## Methods

### Study participants

This report includes results from a subset of participants (*n* = 320) from the National Heart, Lung and Blood (NHLBI) Growth and Health Study (NGHS) at baseline of the 20-year follow up cohort. The original NGHS examined risk factors for cardiovascular disease in socioeconomically-diverse Black and White girls from childhood (ages 9–10) through young adulthood. Participants were recruited from three regions of the U.S.: Western Contra Costa County in California, Cincinnati, OH and Washington, D.C. who self-identified their race as Black or White. Building on longitudinal data from participants recruited from Western Contra Costa County site (*n* = 887), the current follow-up NGHS cohort assesses their health at ages 37 to 43 years. Participant eligibility and study protocol is described elsewhere. Briefly, participants were eligible to enroll in the study if, at the time of enrollment, they were: 1. Not currently pregnant, 2. Had not given birth, experienced a miscarriage, or had an abortion in the past three months, 3. were not currently living abroad and 4. were not institutionalized or in prison. A total of 624 women (73%) of eligible participants enrolled in this study. Participants who provided written consent for protocol number 2013-11-5774 were enrolled. Informed consent was obtained during the in-person visit for local participants. For participants who had relocated farther than 65 miles from Berkeley, CA, consent forms were mailed and signed consent forms were returned to study staff by mail. This study was approved by the Institutional Review Board at the University of California, Berkeley.

### Participants characteristics

Body weight was measured during the follow-up annual in-person visit at either their home or local clinic. The visit protocol is described elsewhere, but briefly included completion of consents, anthropometric measures and blood draw appointment scheduling. Blood draws were collected for the measurement of fasting glucose and insulin concentrations. Insulin sensitivity was determined by HOMA-IR: glucose (mg/dL) x insulin (uU/mL)/405. HOMA-IR cutpoint was based on the 75% percentile HOMA-IR values of normal weight Black and White women with BMI <25 kg/mg^2^, per recommended cutpoint criteria [43, 44]. Participants with a HOMA-IR <2.5 were characterized as insulin sensitive; HOMA-IR ≥ 2.5 characterized insulin resistance.

### Fecal sample analysis for gut microbiome profiles

A total of 320 women provided fecal samples for the current analysis. All study participants were given the choice to complete a stool sample using a Ubiome collection kit (UBiome, San Francisco, CA). Participants were eligible to complete a stool sample after they enrolled in the study, completed their consent forms with study staff, and completed the baseline survey. Samples were collected between March 2019 and September 2019.

During the visit, remote or in-person, each participant was provided a Ubiome sample kit that was labeled with their unique kit ID and specific instructions for use. The “Stool Kit Instruction Insert” provided both written and visual instructions on how to use the Ubiome stool sample it. Study staff also reviewed the instructions in detail with the participants and answered any questions. Participants were instructed on sample collection hygiene and sterility including avoiding contamination of collection swab to anything other than the fecal sample (e.g. fingers, hair, floor, etc.), and avoiding the use of chlorinated pool or hot tub, or engaging in sexual activity less than 8 hours prior to sample collection. Participants were also asked by study staff if they had taken antibiotics recently. If antibiotics were taken, the participant was instructed to wait three months from the date that they ended their antibiotic course to permit gut microbiome recovery before collecting the sample. Research suggests that gut microbiome mostly recovers between 1 to 1.5 months [45, 46]. Participants were also asked to disclose if they had any gastrointestinal conditions, as inflammatory bowel syndrome, inflammatory bowel disease [47] as well as other conditions that can influence the gut microbiome. After completing the sample, participants used a pre-packaged envelope to send their sample directly to Ubiome for processing.

Processing of UBiome fecal samples have been previously described [48]. Briefly, samples were lysed by bead-beading and DNA was extracted in a class 1000 room using guanidine thiocyanate silica column-based purification method. Universal primers containing Illumina tags and barcodes were used for polymerase chain reaction (PCR) amplification of 16S rRNA genes. PCR products were pooled, column purified, and size-selected through microfluidic DNA fractionation [49]. Sequencing was performed in a pair-end modality on the Illumina NexSeq 500 platform rendering 2 x 150 bp pair-end sequences.

After quality control of the raw sequence files, to ensure each sample had paired end reads information, 258 samples were processed using the dada2 1.14.1 [50], in R v3.6.3 allowing for 2 expected errors, 0 Ns and a minimum Q score of 2. Taxonomy was inferred using the DECIPHER package and the silva 132 database. Taxonomic ranks were as follows: kingdom, phylum, class, order, family, genus and species. After removal of standard contaminant taxa, we filtered the data to only include taxa with a prevalence greater than 5%. Taxonomic reduction (tip_glom from phyloseq) was performed by collapsing taxa closer than 0.1 on a phylogenetic tree built using FastTree (v 2.1.10). The final analysis included 168 participant samples an additional prevalence filtering of 30%.

Data from this study are available at datadyrad [51].

### Statistical analysis

Descriptives: A univariate analysis determined unequal distribution for BMI, fasting glucose, fasting insulin and HOMA-IR. To test for differences between groups in these outcomes, we used nonparametric statistical analysis with Wilcoxon test. Difference in age was determined by general linear model. Differences in distribution of insulin sensitivity status between race groups were determined by chi-square test.

Microbiota analyses: Our analyses focused on identifying potential differences in gut health based on phylum and family relative abundance, and diversity measures (alpha and beta). The data manipulation and statistical analysis of bacteria taxonomy, such as alpha and beta diversity, was done using phyloseq 1.30.0 [52] and plotted using ggplot2.

Alpha diversity, a measure of diversity within each sample, was measured by three measures: Shannon Index, Simpson, or Chao. The dataset was rarefied to 10,000 reads and non-normally distributed variables were log-transformed. Some samples were discarded through rarefaction set at 10,000 reads for the alpha diversity measures. Shannon and Simpson Indices both weigh relative microbial community richness based on amplicon sequence variant (ASV) and evenness of representation within a sample [53, 54]. The Chao Index determines richness calculating the expected diversity of ASV based on the presence of all species present within a sample [55]. To measure beta diversity, a measure of diversity between groups, we used the principal coordinate analysis (PCoA) from Bray-Curtis dissimilarity distances available through the phyloseq R package.

Microbiota analyses by race and insulin resistance: To determine differences in alpha diversity and taxonomy by race, we used a general linear model with tukey post-test for comparisons between groups by race (Black vs White). Based on prior studies demonstrating the significant effect of BMI on bacteria profiles, all models included BMI as a covariate. A second model included both BMI and insulin sensitivity status as covariates (insulin sensitive (IS) or insulin resistant (IR)) and tested for an interaction between race and insulin sensitivity status (race*HOMA-IR status). Post-hoc analyses tested for differences between groups categorized by race and insulin sensitivity classification (Black insulin sensitive (IS Black); Black insulin resistant (IR Black); White insulin sensitivity (IS White); White insulin resistant (IR White)). All reported p-values include adjustments for BMI. Differences in relative abundance at the phylum level were adjusted for Bonferroni corrections based on 7 observations at the phylum level and 30 observations at the family level.

## Results

Participant characteristics are listed in **Table 1**. Women were of similar age, however BMI and fasting insulin were significantly greater in Black women (p = 0.0002 and p = 0.0002, respectively). Insulin sensitivity, estimated by HOMA-IR, was significantly lower in Black women compared to White women (p = 0.0002). The distribution of insulin sensitivity status between race groups were significantly different (p = 0.03). Both groups had a greater proportion of insulin sesntive versus insulin resistant women. However, 46% of Black women were insuln resistance while only 30% of White women were insulin resistant (**Table 1**).

**Table 1:** Participant characteristics.

	All	White	Black	
	(*n* = 168)	*(n* = 74)	(*n* = 94)	
	mean ± SD or % (n)	mean ± SD or % (n)	mean ± SD or % (n)	*p* ^race^
Age	39.1 ± 1.1	39.0 ± 0.92	39.1 ± 1.1	0.30
BMI (mg/kg^2^)	31.3 ± 8.8	29.0 ± 8.0	33.2 ± 9.0	0.0002
Fasting glucose (mg/dL)	95.0 ± 30.1	92.0 ± 14.4	97.4 ± 38.0	0.25
Fasting insulin (mg/dL)	11.9 ± 8.9	9.8 ± 6.5	13.5 ± 10.1	0.0002
HOMA-IR	3.0 ± 2.9	2.4 ± 2.1	3.4 ± 3.4	0.0002
Insulin sensitive (%/n)	61(103)	70 (52)	54 (51)	0.034
Insulin resistant (%/n)	39 (65)	30 (22)	46 (43)	

*p*^race^ = p-value determined by general linear model with race as categorical variable.

After sequence quality control, sample selection and rarefaction, a total of 168 samples (*n* = 94 Black and *n* = 74 White women) remained with 332 taxa at the phylum, family and genus levels. However, after filtering for prevalence of bacteria, only 57 taxa remained: 7 phyla, 30 families and 20 genera. Here, we report only diversity measures, phylum and family relative abundances. Data at the genus level was undetected in several samples resulting in low yield and insufficient sample size for comparison.

### Bacteria differences at the phylum level

Seven phyla were detected in our participants: Actinobacteria, Firmicutes, Bacteroidetes, Fusobacteria, Epsilonbacteraeota, Verrucomicrobria, and Proteobacteria. The two most abundant phyla in our sample were *Firmicutes* and *Bacteroidetes*, constituting 55% and 34% of total bacteria in all participants. However, there were no differences in the relative abundance of *Firmicutes* or *Bacteroidetes*, nor the *Firmicutes/Bacteriodetes* ratio by race (p = 0.85, p = 0.09, and p = 0. 16, respectively). Including HOMA-IR in the model and testing for an interaction with race did not improve these outcomes. The interactions between race and insulin sensitivity status did not reach significance for *Firmicutes* (p = 0.10) or *Bacteroidetes* (p = 0.07).

**Fig 1:**
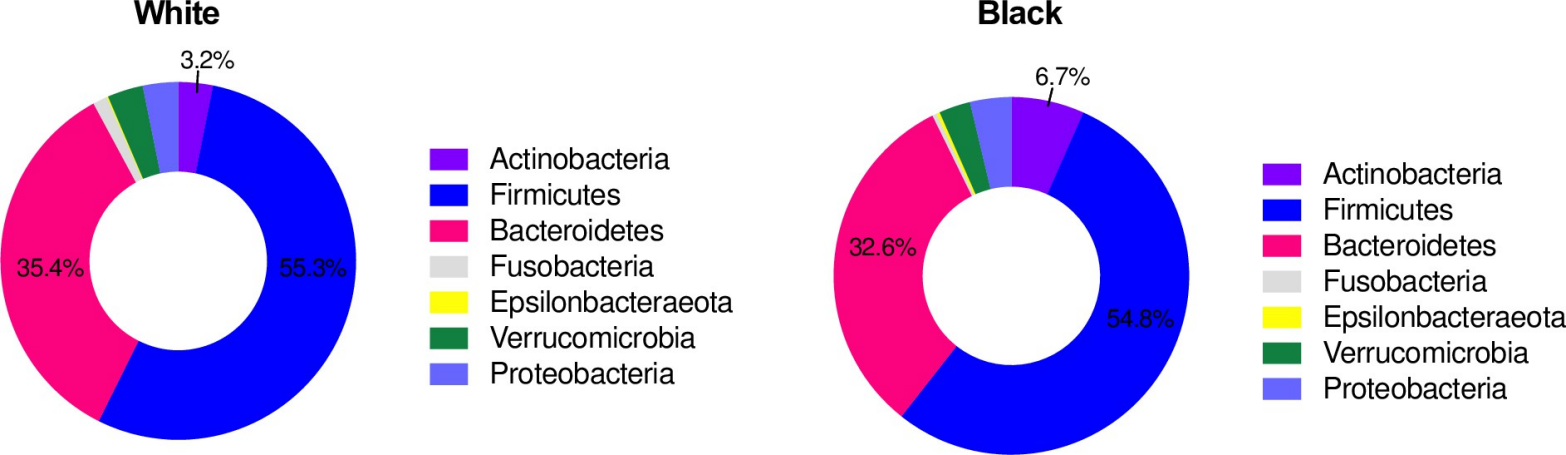
Comparison of relative abundance of bacteria in fecal samples by phylum in White and Black women. Relative abundance was measured by 16S rRNA gene PCR and sequencing.

Black women in our sample had approximately twice the proportion of *Actinobacteria* (6.8%) compared with White women (3.2%) (p = 0.003; p = 0.02 after Bonferroni corrections, **Fig 1**). Race differences remained significant after adjusting for insulin sensitivity status (p = 0.006, or p = 0.04 after Bonferroni). There was no interaction between race and insulin sensitivity status in this model (p = 0.50).

**Fig 2:**
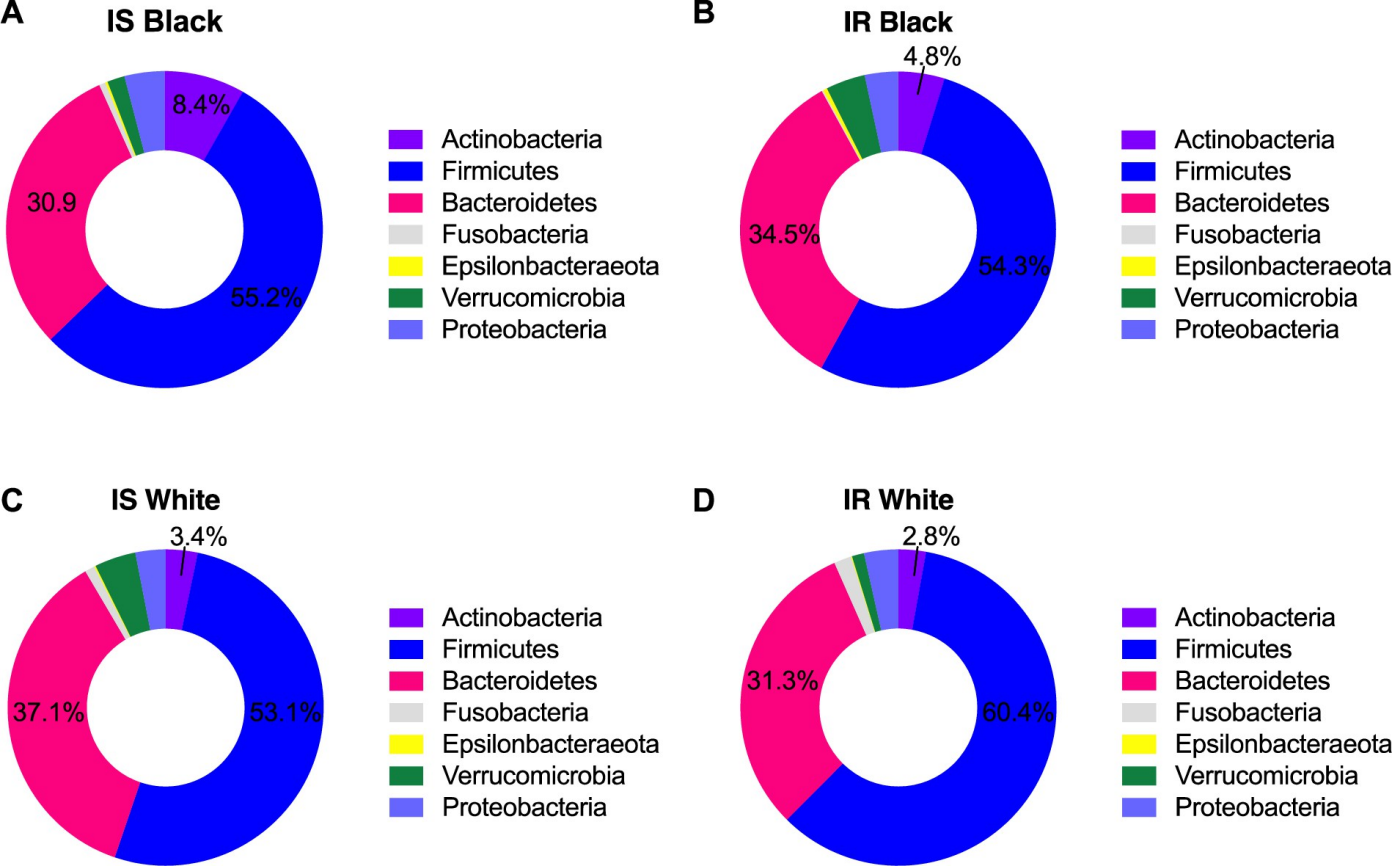
Relative abundance of bacterial phylum in fecal samples by race and insulin sensitivity status. Relative abundance was measured by 16S rRNA gene PCR and sequencing.

The relative abundance of the phyla *Verrucomicrobia* and *Proteobacteria* represented a mean of 3.1% and 3.6% of all phyla, respectively. There were no race differences in *Proteobacteria* (p = 0.22). BMI significantly contributed to the variability between groups for *Proteobacteria* (BMI, p-0.02); removing BMI from the model resulted in a p-value of 0.08. We also did not observe differences by race alone for *Verrucomicrobia* (p = 0.22). Adding insulin sensitivity status to the model improved the statistical values for race comparisons (p = 0.07), but this did not reach significance. We observed a significant race x insulin sensitivity status interaction (p = 0.008) for *Verrucomicrobia*. There was a 4 fold higher level of *Verrucomicrobia* in Black women with insulin resistance vs White women with insulin resistance (4.1% vs. 1.2%, p = 0.02; p = 0.14 after multiple corrections). We did not observe any statistically significant differences in the relative abundance of *Fusobacteria* or *Epsiolobacteria* between groups. Phylum distribution by race and insulin sensitivity status is depicted in **Fig 2**.

### Race comparisons of bacteria at the family level

**Fig 3:**
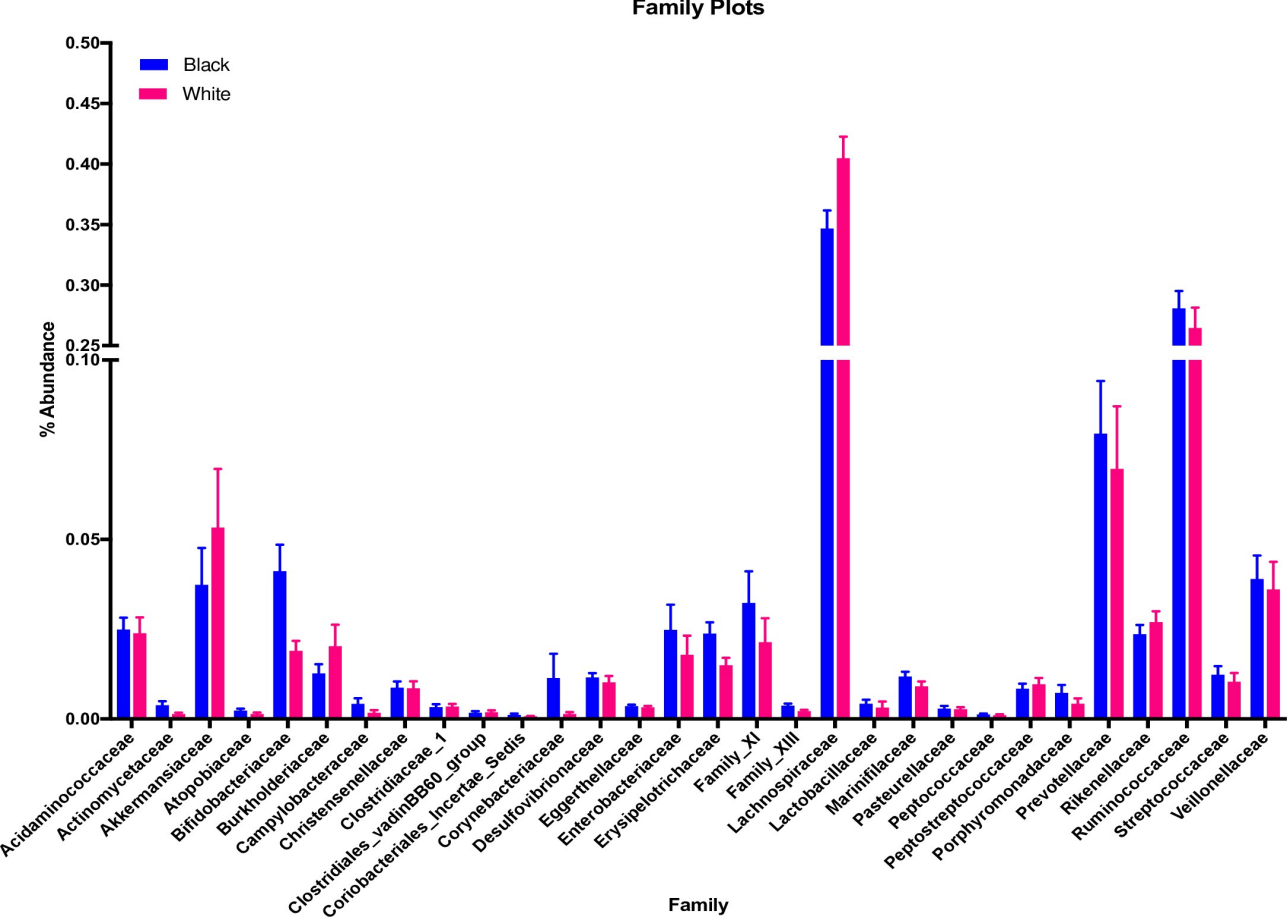
Family abundance by race. Bar graph of the % abundance of 30 identified bacterial families in 168 Black (n = 94) and White (n = 74) women.

We detected 30 different taxa at the family level (**Fig 3**) and only 20 taxa at the genus level. We found that due to low yield of bacteria at the genus level, differences were statistically significant at the family level but not at the genus level. Therefore, only family was included in the final analysis.

**Fig 4:**
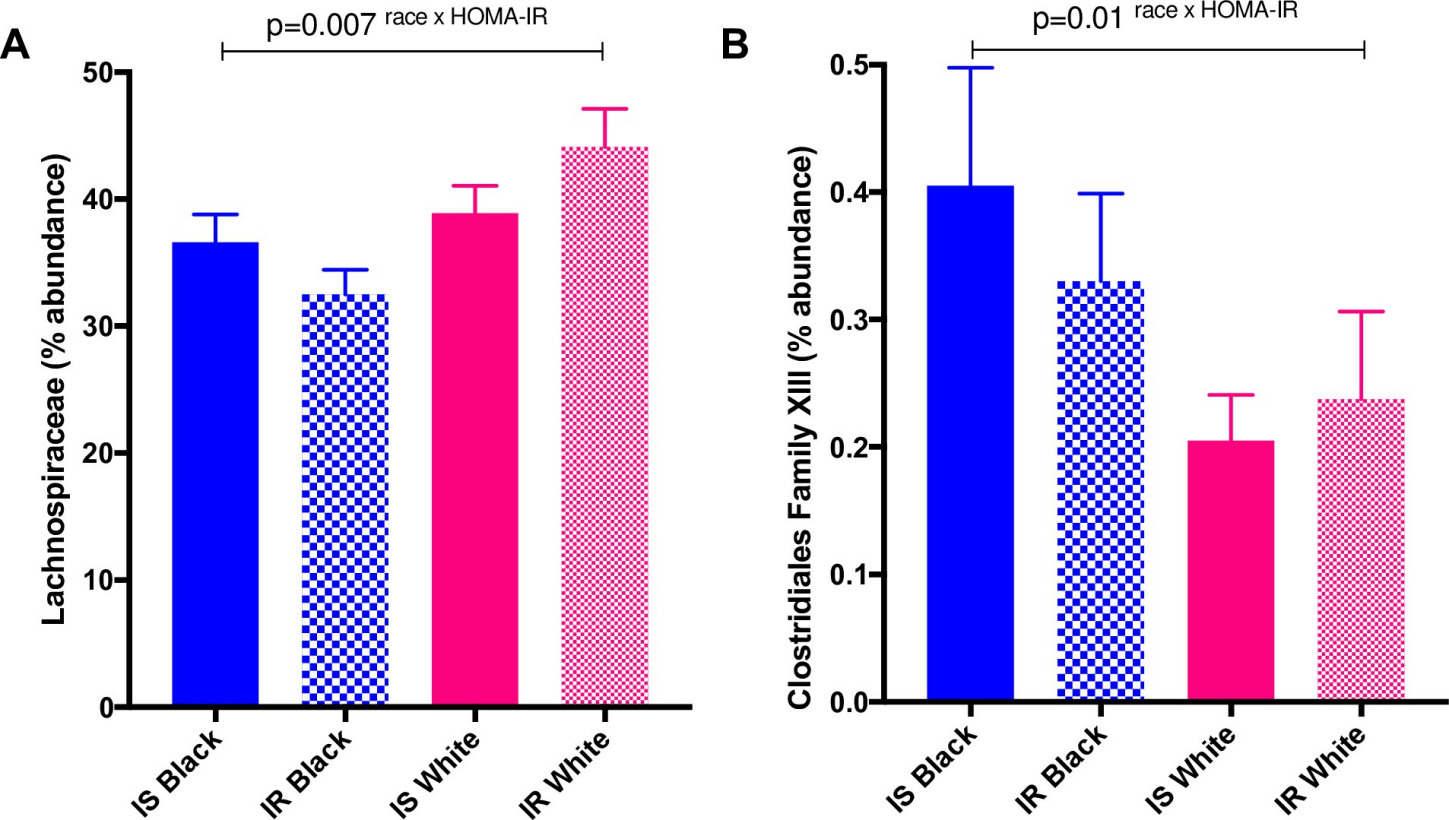
Relative abundance of A) *Lachnospiraceae* and B) *Clostridales Family XIII* in fecal samples by race and insulin sensitivity status. Statistical significance determined by a general linear model testing for race x HOMA-IR interaction; significance p<0.05. Relative abundance was measured by 16S rRNA gene PCR and sequencing.

We did not observe any significant differences by race for any of the bacteria we identified at the family level. Although we did not observe any differences by race alone for families *Clostridiales Family XIII* (p = 0.45) and *Lachnospiraceae* (p = 0.85), there were significant interactions between race and insulin sensitivity status for these two families (*Clostridiales Family XIII* (p = 0.01) and *Lachnospiraceae* (p = 0.007) (**Fig 4**)). There was twice the relative abundance of *Clostridiales Family XIII* in insulin sensitive Black women in comparison with insulin sensitive White women, although this did not reach significance (p = 0.07). There were no differences by race amongst insulin resistant women. *Lachnospiraceae* tended to be greater in insulin sensitive Black women as compared with insulin resistant Black women (p = 0.06). We did not observe differences amongst White women (p = 0.59). Inclusion of insulin sensitivity status in the model did not change significance values for race-only comparisons in either family (*Family XIII* (p = 0.15) and *Lachnospiraceae* (p = 0.64)).

### Gut bacteria diversity

**Fig 5:**
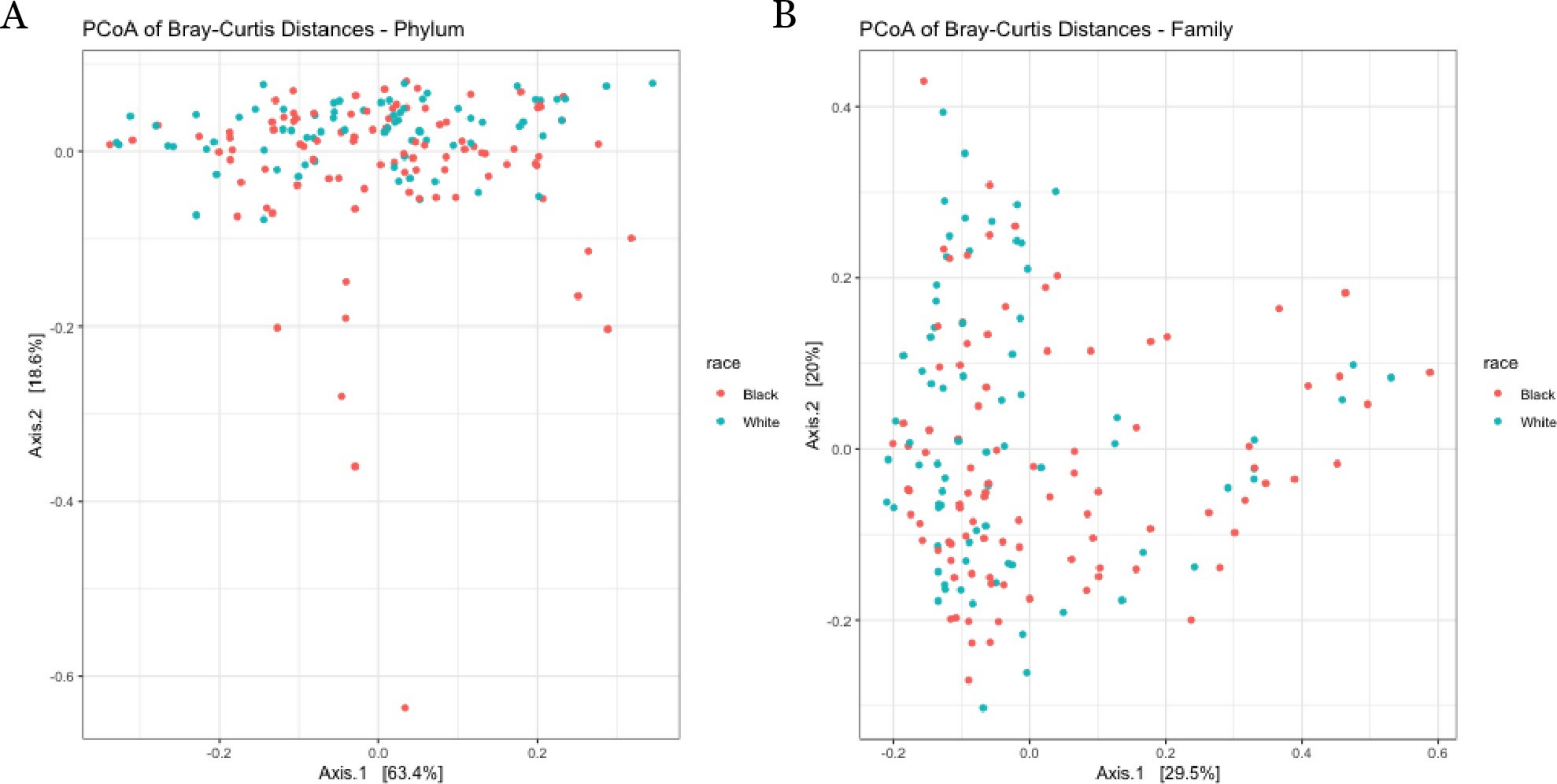
Principal coordinate analysis (PCoA) of relative abundance of bacteria in fecal samples by A) phylum and B) family in White and Black women.

**Fig 6:**
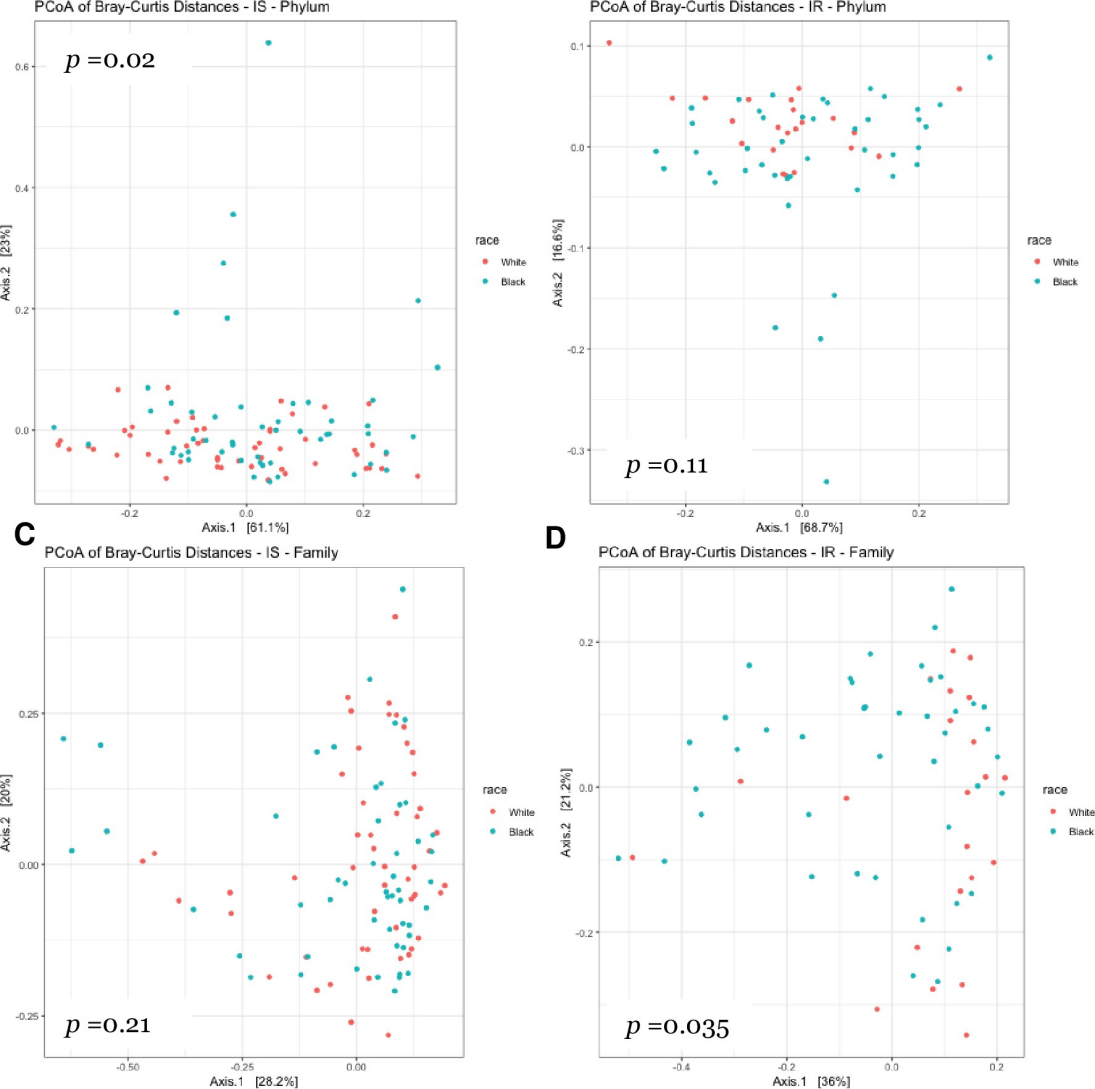
Race comparisons of beta diversity of bacteria by insulin sensitivity status. Principal coordinate analysis (PCoA) of relative abundance of taxa at the phylum level amongst A) insulin sensitive women (*n =* 101) and B) insulin resistant individuals (*n =* 64). Principal component of analysis (PCoA) of relative abundance of taxa at the family level amongst C) insulin sensitive women (*n =* 101) and D) insulin resistant individuals (*n =* 63). Relative abundance was measured by 16S rRNA gene PCR and sequencing.

Beta diversity by race at the phylum and family levels are depicted in the principal coordinate analysis (PCoA) plots in **Fig 5A and 5B**. Differences in beta diversity by race at the phylum level did not reach significance (p = 0.066). However, at the family level, beta diversity was significantly different by race (p = 0.033). **Fig 6** depicts PCoA plots when participants were categorized based on both race and insulin sensitivity status (IS or IR). Including insulin sensitivity status in the model resulted in significant differences in beta diversity at the phylum level (p = 0.038). This was likely due to differences by race amongst insulin sensitive individuals (p = 0.02). In contrast, at the family level, significant differences in beta diversity were observed only amongst insulin resistant individuals (p = 0.035).

Alpha diversity by Shannon Index, Simpson, or Chao did not differ between Black and White women (p = 0.17, p = 0.23, p = 0.23). Adding additional adjustments for age and insulin sensitivity did not change the results (Shannon Index, p = 0.20; Simpson, p = 0.27; or Chao, p = 0.22).

## Discussion

To our knowledge, this is the first investigation to report differences in gut bacteria by insulin sensitivity status in Black versus White women, and only the third study examining gut microbiota between Black and White women in the US. Our study is also the largest conducted to date and the first to focus on premenopausal Black and White women. Since few studies have characterized microbial communities in women with and without insulin resistance, we sought to explore the gut microbiome as one potential mechanism explaining disproportionately higher insulin resistance in Black women. Indeed, in our sample as well, there was a higher proportion of Black women with insulin resistance compared to White women (46% vs. 30%). Our analyses found that the gut microbiome differed both by race alone and when stratified by insulin sensitivity status, at the phylum and family levels. Specifically, we found significant differences in beta diversity and *Actinobacteria* by race, that were further explained by insulin sensitivity status.

### Findings independent of obesity

Consistent with epidemiological findings [56], Black women in our cohort presented higher BMI as compared with White women. Therefore, in order to determine race differences independent of obesity, all analyses included adjustments for BMI. An important finding in our study is the significantly greater relative abundance of phylum *Actinobacteria* in Black women. Our finding supports previous research demonstrating greater *Actinobacteria* in obese compared to lean individuals [57]. However, this is in contrast to a study by Yang and colleagues that found *Actinobacteria* to be lower in relative abundance in the oral microbiome of Blacks compared to Whites [58]. These opposing results may simply reflect differences in the type of biospecimen collected (e.g. oral vs fecal) [59].

Further analyses in our study found that when comparing race differences based on insulin sensitivity status, the presence of greater *Actinobacteria* in Black women as compared with White women was only observed amongst insulin resistant individuals; there were no racial differences amongst insulin sensitive women. This suggests differential pathways through which *Actinobacteria* either contributes to the development of insulin resistance or is a characteristic of insulin resistance in Black women.

Obesity remains a predominant driver of cardiometabolic disease, and there is evidence that greater *Actinobacteria* characterizes the obese state in Black and White adults [57]. However, in our study, adjusting for BMI did not affect any observed differences by race and insulin sensitivity status, indicating that other factors (e.g. inflammation, diet, psychosocial, discrimination) may explain our observations. Currently, only one study has examined social factors as they relate to race differences in the microbiome and found that in young, healthy, Black women *Bifidobacterium* [the predominant genus under the *Actinobacteria* phylum] was positively associated with psychological stress [41]. This deserves further examination.

### Potential role of inflammation

A greater HOMA-IR score in Black women in our cohort was likely associated with greater inflammation [5, 60]. We predicted that greater HOMA-IR values in NGHS Black women may be associated with a greater relative abundance of Gram-negative bacteria that derive the production and secretion of pro-inflammatory endotoxin, lipopolysaccharide (LPS). Increases in LPS are caused by a reduction in intestinal barrier function in which tight junctions are damaged, resulting in leakage of LPS from Gram-negative bacteria [61]. However, *Actinobacteria* is a Gram-positive bacteria and some recent studies have found a beneficial role of this phylum in maintaining intestinal barrier function, thereby preventing an increase in circulating LPS [62]. This is in contrast to other studies that linked greater *Actinobacteria* to insulin resistance [63], as well as a reduction in proteins responsible for maintaining intestinal barrier function [25]. There are also positive associations between *Actinobacteria* and inflammatory cytokines TNF-alpha, IL-1b and IL-6 [25]. These cytokines are commonly elevated in Black men and women [5, 64, 65], thus the greater relative abundance of *Actinobacteria* in Black women in our study may be promoting inflammation and insulin resistance in this group.

### Family differences (Lachnospiraceae and Clostridiales Family XIII)

In order to assess the gut microbiome beyond just the phylum level, we analyzed taxa at the family level as well. Interestingly, only two families, *Lachnospiraceae* and *Clostridiales Family XIII* (both from the *Firmicutes* phylum) showed significant interactions by race and insulin sensitivity. The interactions for *Clostridiales Family XIII* appear to be driven by race differences only in the insulin sensitive group, whereas differences in *Lachnospiraceae* appear to be driven by insulin sensitivity status only in Black women. We observed a lower relative abundance of *Lachnospiraceae* in insulin resistant compared to insulin sensitive Black women, but there were no differences by insulin sensivity status in White women. A previous study in Ghanian women found that lower relative abundance of *Lachnospiraceae* corresponded to greater insulin levels and HOMA-IR [40]. *Lachnospiraceae* is a short-chain fatty acid (SCFA)-producing bacteria belonging to the *Firmicute*s phylum [66, 67]. Increased SCFA production is linked to obesity, insulin resistance [68] and hepatic fat accumulation [28]. This relationship seems to be largely driven by the SCFA acetate [69]. Butyrate (another SCFA), on the other hand, is thought to be anti-obesogenic, improve insulin sensitivity [70] and protect against of visceral fat accumulation [66]. Interestingly, obesity is and insulin resistance tends to be more prevalent amongst Black women [5, 6], although visceral fat is lower [5, 7]. There may be a link between low *Lachnospiraceae* and greater weight-gain and insulin resistance in Black women, possibly driven by an increased production of acetate. *Lachnospiraceae’s* role in protecting against visceral fat remains unclear in Black women, since this populations tends to have lower visceral fat, regardless of insulin sensitivity status [7]. *Clostridiales Family XIII* on the other hand, only differed by race amongst women who were classified as insulin sensitive. This was an unexpected finding given the associations between low relative abundance of *Clostridiales Family XIII* and insulin resistance [31]. However, its role in the development of insulin resistance has not yet been identified. A longitudinal analysis will help us to better understand the potential roles of *Lachnospiraceae* and *Clostridiales Family XIII* in the development of insulin resistance in Black and White women.

### Alpha and beta diversity

Beta diversity differed between Black and White women, corrorborating findings from smaller studies [40–42]. Importantly, race or ethnic differences in beta diversity may reflect differences in fiber intake. In Black and White postmenopausal women, differences in beta diversity disappeared after a flaxseed diet-controlled intervention [42]. In a study comparing U.S. Black women to Ghanaian Black women, divergent beta diversity reflected significant differences in fiber intake (lower in U.S.Black women) [40]. These studies demonstrate that race/ethnic differences in gut microbiome profiles are likely a reflection of environmental influences such as diet, rather than genetics.

Previous studies have demonstrated lower alpha diversity with obesity and insulin resistance [30, 31]. Alpha diversity, that is diversity within each individual, is in some cases seen an indicator of gut health. Therefore, we hypothesized that alpha diversity would be lower in Black women as compared to White women, coinciding with greater prevalence of insulin resistance in the Black women in our cohort. Surprisingly, we did not observe any differences in alpha diversity, but our results support previous studies compared Black and White women of similar sample size and BMI [41]. Prior studies demonstrating negative associations between alpha diversity and features of cardiometabolic disease did not examine potential sex differences. The opposing result displayed by our study and that of Carson *et al*. [41] emphasize the importance of investigating both race and sex differences in order to better understand the role of gut bacteria in promoting or preventing insulin resistance in diverse populations.

Characterizing the gut microbiome in Black women has the potential to better our understanding of the development of cardiometabolic disease health disparities in this population. Here, we have demonstrated in a cross-sectional analysis that microbial communities differ by race and insulin sensitivity status, suggesting a role of gut bacteria in the pathogenesis of insulin resistance development in women of different race and ethnicities. However, we should not be remiss in noting that profiling gut bacteria by race and ethnicity should not be interpreted as a reflection of genetic ancesteral or inherent differences. Race is a social construct. Thus, our findings that gut bacteria differ in Black and White women highlights the potential impact of social determinants of health on gut bacteria. As researchers have pointed out, the strong influence of race and ethnicity in the Human Microbiome Project must be re-framed in the context of the historic and sociocultural influences rather than simply instrinsic biological differences based soley on race or ethnicity [71]. Possible explanations for race differences will need to be further explored in future studies that include social, environmental and behavioral factors such as diet and psychological stress, as well as examination of pro-inflammatory markers. All of these factors have been shown to alter the gut microbiome.

### Study strengths

Strengths of our study include the use of fecal samples collected from Black and White women in the National Health and Growth Study (NGHS) and the ability to examine microbiome differences based on insulin sensitivity. This pilot study was not without limitations. First, samples were only collected at one time point, thus a longitudinal analysis we not possible. Secondly, examination of lower taxonomy can be more informative in understanding the mechanisms through which gut bacteria may affect metabolic pathways; for example, by linking specific bacteria to the metabolites they produce [18, 35, 72]. Characterization of the gut microbiome at the family and genus levels and their associated metabolites before and after the transition of insulin resistance would uncover the role of gut bacteria in the mechanisms of insulin resistance development in Black women. Snyder and colleagues have conducted a thorough multi-omics study to identify microbial, metabolite and other molecular signatures specific to the development of insulin resistance [20], although analyses by sex or race/ethnicity were not conducted. A third limitation is the use of HOMA-IR to characterize individuals as insulin sensitive or insulin resistance. This simple metric of insulin sensitivity is often utilized in population studies due to its feasibility [73], compared to other more informative metrics like the oral glucose tolerance test, or the gold standard, hyperinsulinemic euglycemic clamp. HOMA-IR is not ideal for the determination of whole-body insulin sensitivity in Blacks [7, 74], thus a longitudinal follow-up study in NGHS women should consider inclusion of an oral glucose tolerance test to understand the potential link between race differences in glucose regulation and gut bacteria. Clinical studies utilizing techniques like the clamp method to measure hepatic insulin sensitivity should consider including stool collection to explore the connection between gut bacteria and race/ethnic differences in tissue-specific insulin resistance (e.g. hepatic, muscle or adipose). Hepatic and whole body insulin resistance tend to be higher in Blacks [75–77], but evidence for adipose insulin resistance is inconclusive [78, 79]. Hepatic insulin resistance may be lower in Black as compared with white women [75] and the gut-liver axis may prove to be one mechanism by which reduced insulin sensitivity is more prevalent in this group [75–77, 80]. Lastly, we did not adjust for the effects of diet, psychological stress, geographical location, or inflammation, which may link racial/ethnic and cultural differences to environmental factors driving differences in microbial communities.

## Conclusion

Our pilot study demonstrates race differences in beta diversity at the family level, plus greater abundance of *Actinobacteria* (phylum) and lower *Lachnospiraceae* (family) abundance in insulin resistant Black women. A follow-up longitudinal investigation is needed in order to better understand the significance of these findings with the inclusion of social determinants of health as potential mediators. Since this is a cross sectional study, we cannot determine causal directions or bidirectionality of the relationships observed. However, it is possble that social and environmental factors associated with Black race contribute to a unique microbiota profile, that in turn contributes to inflammation and insulin resistance, independent of obesity.
